# Current crowding mediated large contact noise in graphene field-effect transistors

**DOI:** 10.1038/ncomms13703

**Published:** 2016-12-08

**Authors:** Paritosh Karnatak, T. Phanindra Sai, Srijit Goswami, Subhamoy Ghatak, Sanjeev Kaushal, Arindam Ghosh

**Affiliations:** 1 Department of Physics, Indian Institute of Science, Bangalore 560 012, India; 2 Tokyo Electron Ltd, Akasaka Biz Tower, 3-1 Akasaka 5-Chome, Minato-ku, Tokyo 107-6325, Japan; 3 Centre for Nano Science and Engineering, Indian Institute of Science, Bangalore 560 012, India

## Abstract

The impact of the intrinsic time-dependent fluctuations in the electrical resistance at the graphene–metal interface or the contact noise, on the performance of graphene field-effect transistors, can be as adverse as the contact resistance itself, but remains largely unexplored. Here we have investigated the contact noise in graphene field-effect transistors of varying device geometry and contact configuration, with carrier mobility ranging from 5,000 to 80,000 cm^2 ^V^−1 ^s^−1^. Our phenomenological model for contact noise because of current crowding in purely two-dimensional conductors confirms that the contacts dominate the measured resistance noise in all graphene field-effect transistors in the two-probe or invasive four-probe configurations, and surprisingly, also in nearly noninvasive four-probe (Hall bar) configuration in the high-mobility devices. The microscopic origin of contact noise is directly linked to the fluctuating electrostatic environment of the metal–channel interface, which could be generic to two-dimensional material-based electronic devices.

## Abstract (web summary)

The performance of graphene field effect transistors is adversely affected by fluctuations in the electrical resistance at the graphene/metal interface. Here, the authors unveil the microscopic origin of such contact noise, highlighting the role of current crowding.

The wide spectrum of layered two-dimensional (2D) materials provides the opportunity to create ultimately thin devices with functionalities that cannot be achieved with standard semiconductors. The simplest of such devices is the field-effect transistor (FET). There are several factors that determine the performance of an FET, the key among them being the dielectric environment, quality of the metal–semiconductor contact and the level of low-frequency 1/*f* noise. Over the past few years, there has been tremendous progress in creating high-mobility, atomically thin FETs through a combination of low-resistance ohmic contacts^1^,^2^,^3^,^4^ and strategies for encapsulation^3^ of the active channel. However, there exists no consensus on the factors that determine the magnitude of the 1/*f* noise, which is known to degrade the performance of amplifiers, or introduce phase noise/jitter in high-frequency oscillators and converters^5^. Noise is especially detrimental to the performance of nanoscale devices and may cause variability even in ballistic transistor channels, where it has been suggested to arise from slow fluctuations in the electrostatic environment of the metal–semiconductor contacts^6^

Even for the widely studied graphene FET, it is still unclear what the dominant contribution is to the 1/*f* noise. Conflicting claims exist, where some studies attribute the 1/*f* noise in graphene transistors primarily to noise generated within the channel region^7^,^8^,^9^, whereas other investigations indicate a strong contribution from the contacts^10^,^11^,^12^. This distinction has remained elusive to existing studies^7^,^8^,^9^,^10^,^11^,^12^,^13^,^14^,^15^,^16^,^17^,^18^,^19^,^20^,^21^ because of the lack of a microscopic understanding of how processes characteristic to the metal–graphene junctions, in particular the current-crowding effect^22^,^23^,^24^,^25^,^26^,^27^, has an impact on the nature and magnitude of 1/*f* noise.

Fundamentally, current crowding is an unavoidable consequence of resistivity mismatch at the metal–semiconductor junction, where the injection and/or scattering of charge carriers between the semiconductor and the metal contact is restricted only close to the edge of the contact, over the charge transfer length *L*_T_^22^,^28^. Photocurrent measurements^29^,^30^,^31^ and Kelvin probe microscopy^32^,^33^ at the graphene–metal interface have already indicated the presence of current crowding with *L*_T_∼0.1–1 μm (refs 24, 25, 34). Restricting the effective current injection area leads to greater impact of local disorder kinetics, and hence larger 1/*f* noise^35^. For graphene–metal interfaces, the scenario is more complex than a typical metal–semiconductor junction, since it is known that metals such as Cr, Pd and Ti react to form metal carbides with graphene, altering the structural properties and causing strong modifications in its energy band dispersion^34^,^36^. While it is clear that current crowding and the characteristics of the metal–graphene junction directly influence the contact resistance^25^,^29^,^34^,^36^,^37^,^38^,^39^,^40^,^41^,^42^, how these factors have an impact on the noise originating at the contacts (contact noise) is still not known.

In this work we study a series of graphene FETs with different mobilities, substrates and contacting configurations to demonstrate that electrical noise at the metal–graphene junction can be the dominant source of 1/*f* noise in graphene FETs, especially for invasive contacting geometry, where the probe contacts lie directly in the path of the current flow. The contact noise was found to scale as 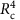, where *R*_c_ is the contact resistance, in all devices and at all temperatures. While the noise magnitude is determined by the fluctuating charge trap potential at the oxide substrate underneath the metal contacts, a simple phenomenological model unambiguously attributes the scaling to the current-crowding effect at the metal–graphene junction. In view of the recent observations of contact noise^43^,^44^ and current-crowding effect in molybdenum disulphide (MoS_2_) and black phosphorus FETs^26^,^27^, many of the results and concepts developed in this paper can be extended to other members of 2D semiconductor family as well.

## Results

### Characterization of Au-contacted graphene

**Figure 1:**
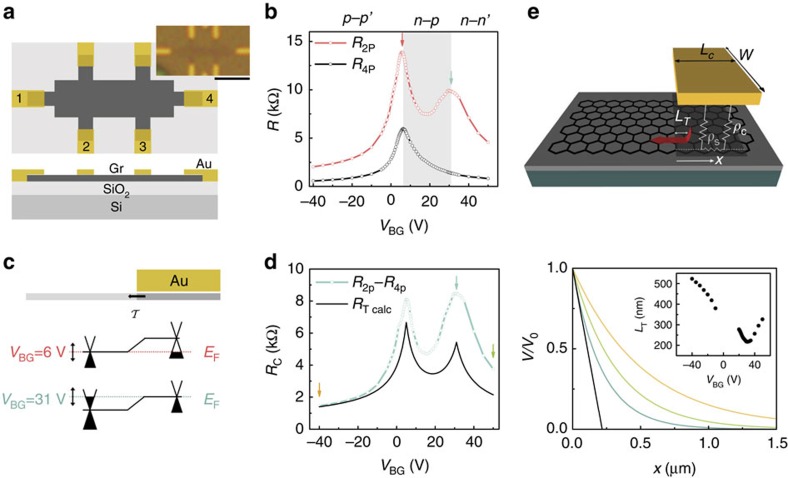
Contact resistance and current crowding in Au-contacted graphene. (**a**) Schematic of the device geometry and contact configuration. Inset shows the optical image; scale bar, 5 μm. (**b**) Resistance as a function of backgate voltage (*V*_BG_) measured in two-probe (*R*_2P_) and the four-probe (*R*_4P_) geometry. (**c**) The Fermi energy of graphene under the Au contacts can be tuned by applying a backgate voltage. (**d**) The difference of the *R*_2P_ and *R*_4P_ gives the measured contact resistance *R*_c_ (blue). The resistance *R*_Tcalc_ calculated for transport across the potential step (black). (**e**) Current injection into graphene occurs within a small length ∼*L*_T_ for a contact length of *L*_c_ (top). Most of the potential drop occurs at the edge of the contact, shown at three *V*_BG_ values (bottom), marked in **d**. Inset shows signification variation in *L*_T_ with *V*_BG_ (data near the main Dirac peak excluded).

We first focus on a single-layer graphene channel on conventional (300 nm) SiO_2_/*p*^++^–Si substrate, etched into a Hall bar shape with surface-contacted Au (99.999%) leads (Fig. 1a). Here we used pure gold contact (without a wetting underlayer of, for example, Cr or Pd) because gold (hole) dopes the graphene underneath without pinning the Fermi energy, or causing substantial modification in the bandstructure^45^,^46^. This allows easy tuning of the doping, and correspondingly the resistance, of the contact region with backgate voltage (*V*_BG_). We measure the two-probe (*R*_2P_=*R*_23,23_) and four-probe (*R*_4P_=*R*_23,14_) resistance and noise as a function of *V*_BG_ between the leads 2 and 3 (suffixes in *R*_*V*+*V*−,*I*+*I*−_ indicate the voltage (*V*^+^, *V*^−^) and current (*I*^+^, *I*^−^) leads). The *V*_BG_ dependence of *R*_2P_ and *R*_4P_ is shown in Fig. 1b. *R*_4P_ shows a slightly asymmetric transfer characteristic, known to occur for asymmetric contact doping^38^, with a single Dirac point at *V*_BG_≈6 V. *R*_2P_, however, shows a second Dirac point at *V*_BG_≈31 V because of the combination of hole doping and weak pinning by Au at the contact region^34^,^38^,^45^,^46^, which divides the transfer behaviour in three parts (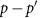, *n*−*p* and 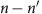), based on the sign of doping in the channel and contact regions. The position of the Fermi level at the two Dirac points is shown in the schematic of Fig. 1c. The observation of double Dirac point in *R*−*V*_BG_ characteristics confirms the structural integrity and gate tunability of the Fermi level of the graphene channel underneath the contact.

### Contact resistance with Au contacts and current crowding

The shift in the local chemical potential by metal contacts results in a change in resistance that contributes to contact resistance, and determines the extent of current crowding at the gold–graphene interface. To compute the contact resistance *R*_c_, we follow the Landauer approach where the net transmission probability *T* across the contact is determined by the interplay of the number of propagating modes in the channel and metal regions (Fig. 1c)^38^. Figure 1d shows the *V*_BG_ dependence of the experimental contact resistance *R*_c_=*R*_2P_−*R*_4P_ (corrected for the resistance of the small region of the probe arms) and that calculated assuming the Dirac-like dispersion and level broadening ≈80 meV underneath the contact and ≈57 meV in the channel (estimated from the experimental transfer characteristics, see Supplementary Note 2 for the full details of calculations). The agreement, both in *V*_BG_ dependence and absolute magnitude (within 50% for all *V*_BG_), indicates that the contact resistance is primarily composed of the resistance *R*_T_ of the graphene layer over the charge transfer length (*L*_T_) underneath the contacts. Owing to mismatch between the resistivities of the metal and graphene, *L*_T_ is significantly smaller than the geometric width *L*_c_ (∼1–1.5 μm) of the metal lead, resulting in the current-crowding effect.

To visualize this quantitatively, we consider the transmission line model where the graphene layer below the contacts is represented with a network of resistors characterized by sheet resistivity *ρ*_T_ (schematic in Fig. 1e). The potential profile in graphene under the metal is then given by^22^,


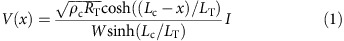


where *I* is the current flowing, *ρ*_c_≈200 Ω μm^2^ (ref. 45) is the specific contact resistivity and 
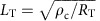
 is charge transfer length from the edge by which 1/*e* of the current is transferred to the metal contact (*W* is the contact width). Taking *R*_T_ as the experimentally observed contact resistance *R*_c_ (Fig. 1d), we calculated the potential drop underneath the contact, normalized to its value at the edge *x*=0, for three gate voltages marked by the arrows in Fig. 1d. The potential drops exponentially over the gate voltage-dependent scale *L*_T_, being minimum (∼200 nm) for the second Dirac point at +31 V where the mismatch between the resistivity of the metal and that of the graphene layer underneath is maximum.

Since 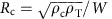 (refs 22, 24) and *ρ*_T_=*R*_T_*W*/*L*_T_, it is evident that the contact noise is essentially the resistance fluctuations in the graphene layer underneath the contact, that is, 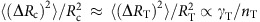, where 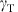 and *n*_T_ are the phenomenological Hooge parameter and carrier density in the charge transfer region, respectively. 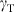 is independent of *n*_T_ and is determined by the kinetics of local disorder induced by trapped charges, chemical modifications and changes in the band dispersion due to hybridization. Assuming a diffusive transport in the charge transfer region with density-independent mobility^46^, the contact noise can be expressed as


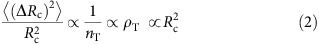


and implies a scaling relation 
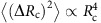
 that can be readily verified experimentally. Note that (1) the scaling is different from that suggested for metal and three-dimensional (3D) semiconductors where the exponent of *R*_c_ is ≈1 for interface-type contacts or ≈3 for constriction-type contacts^35^. (2) Since *n*_T_ is the only gate-tunable parameter^34^,^38^,^39^, the scaling of contact resistance and electrical noise can be dynamically monitored by varying the gate voltage, circumventing the necessity to examine multiple pairs of contacts to isolate the contact contribution to noise. (3) Although the absolute magnitude of the contact noise is device/contact-specific, the scaling of equation (2) is expected to hold irrespective of the geometry, material or chemical nature of the contact (wetting or non-wetting).

### Noise measurement in Au-contacted graphene

**Figure 2:**
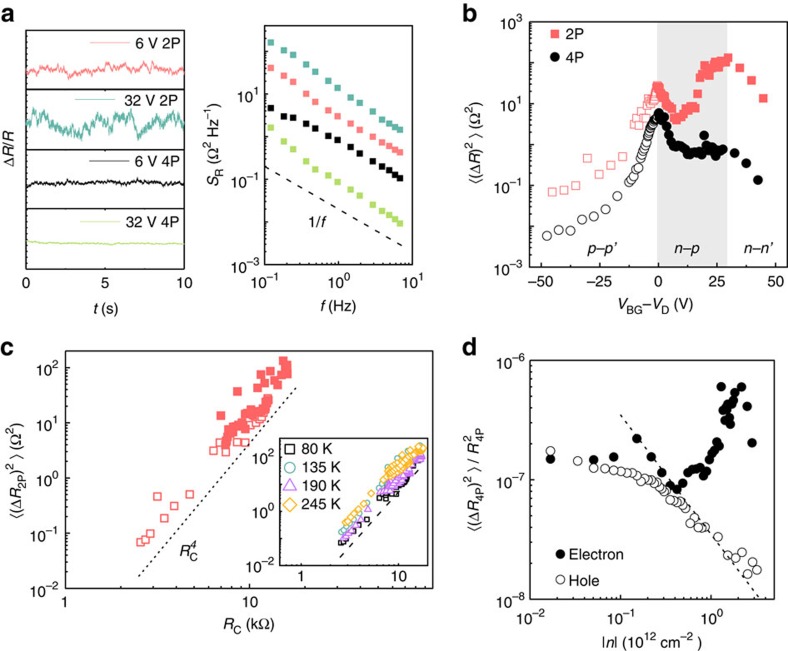
Contact noise in Au-contacted graphene. (**a**) Typical measured time series (left) and corresponding power spectral density *S*_R_ (right) as a function of frequency. (**b**) Noise (variance) as a function of gate voltage in two-probe 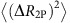 and four-probe 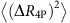 geometry. (**c**)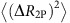 as a function of contact resistance *R*_c_ shows 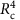 dependence. This dependence is valid for temperatures down to ∼80 K (inset). (**d**) Noise (4p) normalized by the graphene resistance as a function of number density.

Noise in both *R*_2P_ and *R*_4P_ at all *V*_BG_ consists of random time-dependent fluctuations with power spectral density 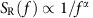 (Fig. 2a), where 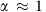 indicates usual 1/*f* noise due to many independent fluctuators with wide distribution of characteristic switching rates. However, to estimate and compare the total noise magnitude, we have evaluated the ‘variance' 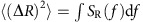, by integrating *S*_R_(*f*) numerically over the experimental bandwidth.

**Figure 3:**
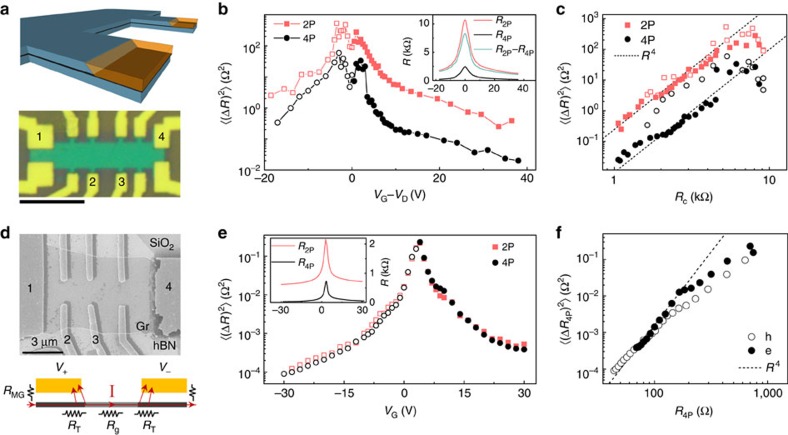
Noise in high-mobility graphene (**a**) Hall bar device with graphene encapsulated by hexagonal BN and contacted by etching the top BN; scale bar, 10 μm in the optical image (bottom). (**b**) Noise variance 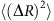 as a function of gate voltage in two-probe and four-probe geometry. Inset shows resistance (*R*_2P_, *R*_4P_ and *R*_c_) as a function of backgate voltage. (**c**) Noise variance 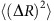 as a function of contact resistance *R*_c_ shows 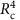 dependence (dotted line) for both two-probe and four-probe measurements. (**d**) Electron microscope image of graphene on BN with invasive linear contacting geometry. Bottom schematic shows the effect of charge scattering, from the region under the contacts, on four-probe measurements as well. (**e**) 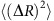 measured in two-probe and four-probe geometry roughly coincide for all gate voltages, indicating that noise generated in the channel is negligible. Inset shows *R*–*V*_G_ characteristics for the device. (**f**) Noise shows *R*^4^ dependence (dotted line) at higher gate voltages indicating the dominant contact contribution.

Figure 2b shows the Δ*V*_BG_ dependence of 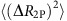 and 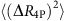, where the maxima in both quantities align well with the Dirac points in *R*_2P_ and *R*_c_ (Fig. 1b,e). The origin of the maximum in noise at the Dirac point is a debated topic, and has often been attributed to low screening ability of the graphene channel to fluctuating Coulomb potential at the channel–substrate interface^7^,^8^,^15^,^16^. Here 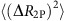 peaks in the *n*–*p* region close to Δ*V*_BG_≈25–30 V, where the density of states in the charge transfer region is low^34^,^38^, indicating contact noise that originates because of poorly screened fluctuations in the local Coulomb disorder. In fact, the noise magnitude at the second peak (Δ*V*_BG_≈30 V) is ∼10 times larger than that at the main Dirac peak, indicating the significantly larger noise where the current crowding is most severe and contact resistance is the largest. Surprisingly, 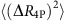 shows a weak increase in this regime as well, suggesting a leakage of the noise at the contacts even in four-probe measurements (discussed in more detail in the context of Fig. 3c and in Supplementary Note 3).

To verify the contact origin of noise, we have plotted 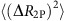 as a function of contact resistance *R*_c_ in Fig. 2c. Remarkably, 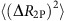 for all *V*_BG_ collapses on a single trace, and varies as 
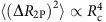
 over four decades of noise magnitude, suggesting that the measured noise in two-probe configuration originates almost entirely at the contacts, which is at least a factor of 10–100 higher than the channel noise 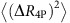 (circles in Fig. 2b). Similar behaviour was observed over a wide temperature range as shown in the inset of Fig. 2c. In order to analyse the channel contribution to noise, 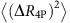 is shown as a function of the carrier density *n* in Fig. 2d. For large hole doping (≳10^12^ cm^−2^), that is, *p–p′* regime where *R*_c_ reduces to ≲1 kΩ, we observed 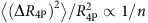 (dashed line), suggesting Hooge-type mobility fluctuation noise in the graphene channel, with a Hooge parameter ∼10^−3^ (refs 8, 11, 13, 47, 48). However, in the *n–n* regime, where the contact contribution is dominant, noise deviates from 1/*n* behaviour.

### Noise in high-mobility graphene hybrids

**Figure 4:**
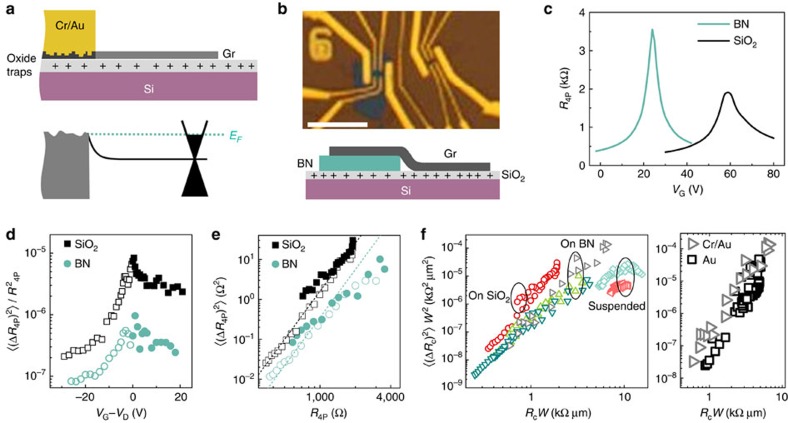
Noise mechanism. (**a**) Schematic of the contact region with metal deposition, and possible effects on the band dispersion and structural properties of graphene underneath. (**b**) Device image and schematic showing a portion of graphene on BN and the rest on SiO_2_. Scale bar, 10 μm. (**c**) Resistance as a function of gate voltage on BN (blue) and on SiO_2_ (black). (**d**) Approximately 5–20 times lower noise in graphene on BN (black squares) than on SiO_2_ (blue circles). (**e**) 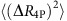 as a function of *R*_4P_ for graphene on SiO_2_ (squares) and for graphene on BN (circles); the dotted lines are *R*^4^. (**f**) Comparison of contact noise in various devices; noise depends strongly on the substrate (left) or the contact material used (right).

The dependence of the noise magnitude with the contacting geometry in the Au-contacted device (Fig. 2) led us to explore the contact contribution to noise in three other device geometries: (1) graphene, encapsulated between two hexagonal boron nitride (BN) layers and etched into a Hall bar, contacted by etching only the top BN (see Methods and Supplementary Note 1), shown in Fig. 3a. (2) Graphene on SiO_2_ and BN substrates (Fig. 3d and Fig. 4b), in surface-contacted linear geometry, where the contacts extend on to the channel region (invasive contacts) and (3) suspended graphene devices that are intrinsically in two-probe contact configuration. A 5 nm Cr underlayer was used with 50 nm Au films as contact material in all these devices. The details of the device fabrication process are given in the Methods section and Supplementary Note 1. Similar to the Au-contacted device, the noise measurements were performed from 80 K to room temperature and no appreciable qualitative difference was observed.

To examine the generality of the 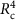 scaling in high-mobility graphene FETs, we first measured both two-probe and four-probe noise in the BN-encapsulated graphene hall bar device, which exhibited room temperature (four probe) carrier mobilities of 58,000 and 35,000 cm^2 ^V^−1 ^s^−1^ in the electron-doped and hole-doped regimes, respectively. The transfer characteristics show only one Dirac point (*V*_D_) for both *R*_2P_ and *R*_4P_ (Fig. 3b inset), as expected for a Cr underlayer^39^. Both 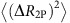 and 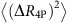 decrease with increasing |*V*_G_−*V*_D_| (Fig. 3b), except over a small region around *V*_D_ where the the distribution of charge in graphene becomes inhomogeneous. Away from the inhomogeneous regime, both 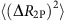 and 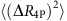 exhibit the 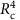 scaling over three decades (Fig. 3c). The 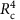 scaling of 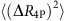 is unexpected, although the suppression 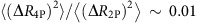 is close to the nonlocal factor ∝ exp[−2*πL*_T_/*W*] for realistic *L*_T_ of ∼400 nm (refs 24, 49), suggesting that this could be a nonlocal effect due to finite dimensions of the voltage leads 2 and 3 (ref. 50; see Supplementary Note 3). This also explains the reduced, but perceptible signature of contact noise in 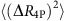 in Fig. 2b,d. It is also interesting to note the drop in contact noise magnitude in the inhomogeneous regime, which could be due to the dominance of McWhorter-type number fluctuation noise^8^,^15^,^16^,^18^,^51^, rather than just mobility fluctuations in the charge transfer region.

The effect of contact noise becomes more severe for invasive surface contacts (leads extending to the current flow path), as demonstrated with a device that has graphene on BN (Fig. 3d). The transfer characteristics show a single Dirac point with carrier mobility ∼35,000 cm^2 ^V^−1 ^s^−1^ (Fig. 3e inset). Strikingly, the magnitudes of 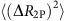 and 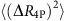 were found to be almost equal over the entire range of *V*_BG_ (Fig. 3e), suggesting that the dominant contribution to noise arises from the charge transfer region underneath leads 2 and 3. To establish this quantitatively, we note that *R*_2P_≈2*R*_MG_+2*R*_T_+*R*_g_ and *R*_4P_≈2*R*_T_+*R*_g_, respectively (see schematics in Fig. 3d), where *R*_MG_ (∼300 Ω) and *R*_g_ are the metal–graphene interface resistance and graphene channel resistance, respectively. Owing to the inseparability of *R*_T_(=*R*_c_) and *R*_g_ within this contacting scheme, we plot 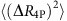 as a function of *R*_4P_ in Fig. 3f. It is evident that 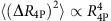 for *R*_4P_≲150–200 Ω, where *R*_g_ is small because of heavy electrostatic doping of the channel. However, for *R*_4P_≳200 Ω, the deviation from the 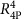 scaling is likely due to finite *R*_g_ that causes *R*_4P_ to overestimate the true *R*_c_. We have observed an *R*^4^ scaling of noise for high-mobility suspended graphene devices as well (see Fig. 4f).

## Discussion

Contact noise at the metal–semiconductor interface has been extensively researched over nearly seven decades^6^,^35^,^43^,^52^,^53^,^54^,^55^,^56^,^57^, and except for a few early models based on kinetics of interface disorder such as adsorbate atoms^53^, the most common mechanism is based on time-dependent fluctuations in the characteristics of the Schottky barrier at metal–semiconductor junctions^53^,^55^,^56^,^57^. The linearity of *I*–*V* characteristics (not shown) and temperature independence of *R*_c_ (see Supplementary Fig. 4) in our devices, however, eliminate the possibility of Schottky barrier-limited transport. An alternative source of time-varying potential is the trapped charge at the SiO_2_ surface^44^,^58^,^59^,^60^,^61^,^62^, which has been suggested to cause contact noise even in ballistic semiconducting carbon nanotube FETs^6^,^63^. The reaction of graphene with metals spontaneously leads to chemical modification (for example, carbide formation) and introduction of defects (see schematic in Fig. 4a). The chemical modification and defect formation can strongly influence the bandstructure of graphene underneath the metal, suppressing the screening of Coulomb impurities. This makes the charge transfer region susceptible to mobility fluctuations because of trapped charge fluctuations in SiO_2_, as indeed shown recently for noise at grain boundaries in graphene^64^.

To verify this, we have fabricated an invasively Cr/Au-contacted device where a single graphene channel was placed partially on BN (thickness ∼10 nm), thus physically separating the channel from the oxide traps^65^, whereas the other part was directly in contact with SiO_2_ (Fig. 4b). The four-probe transfer characteristics (Fig. 4c) confirm that the region of graphene placed on SiO_2_ shows lower carrier mobility (7,500 and 4,000 cm^2 ^V^−1 ^s^−1^ for hole- and electron-doping, respectively) than the corresponding mobility (8,000 and 7,500 cm^2 ^V^−1 ^s^−1^) of the part on BN, as well as strong substrate-induced doping, both of which can be readily understood by the proximity to charge traps at the SiO_2_ surface. Although 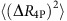 in both parts shows strong peaks at the respective Dirac points (Fig. 4d), it is evident that the normalized noise magnitude in the graphene on SiO_2_ substrate is up to a factor of 10 larger than that on BN, similar to that reported recently^19^,^20^. The scaling 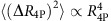 (Fig. 4e) over three decades of noise magnitude, irrespective of the substrate, unambiguously indicates the dominance of contact noise, and that the contact noise in graphene FETs is primarily a result of mobility fluctuations in the charge transfer region due to fluctuating Coulomb potential from local charge traps (predominantly from the SiO_2_ surface).

Finally, in order to outline a recipe to minimize the contact noise in graphene devices, we have compiled the normalized magnitude of specific contact noise 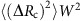 as a function of specific contact resistance *R*_c_*W*, from different classes of devices that were studied in this work. We identify two key factors that have an impact on the contact noise: first, as can be clearly seen in Fig. 4f (left), the specific contact noise is largest for graphene on SiO_2_, lower on devices with graphene on BN and lowest for suspended graphene devices where all SiO_2_ has been etched away from under the graphene channel as well as partially from below the contact region (see Supplementary Fig. 5). Moreover, noise data from all devices with BN as substrate collapse on top of each other, regardless of mobility values, indicating that the separation of contacts from the SiO_2_ traps is the primary factor that determines the noise magnitude rather than the channel quality itself. Second, it can also be seen from Fig. 4f (right) that the device with Cr/Au contacts, which are known to chemically modify graphene^36^,^39^, exhibits higher noise than the device with Au contacts, which is expected to leave graphene intact, despite the fact that the former device has a BN substrate, whereas the later SiO_2_. This highlights the major role of defects under metal contacts in noise generation. Combining these factors leads to the conclusion that minimizing environmental electrostatic fluctuations and developing a contacting scheme that preserves the chemical/structural integrity of graphene will be necessary for ultralow noise graphene electronics.

In conclusion, we have studied electrical noise at the metal contacts in graphene devices with a large range of carrier mobility, on multiple substrates with various device and lead geometries. Using a phenomenological model of contact noise for purely 2D materials, we show that contact noise is often the dominant noise source in graphene devices. The influence of contact noise is most severe in high-mobility graphene transistors. Most surprisingly, we discover the ubiquity of contact noise, which is seen to affect even four-probe measurements in a Hall bar geometry. Our analysis suggests that contact noise is caused by strong mobility fluctuations in the charge transfer region under the metal contacts because of the fluctuating electrostatic environment. A microscopic understanding of contact noise may aid in the development of ultralow noise graphene electronics.

## Methods

### Device fabrication

Graphene and hexagonal BN were exfoliated on SiO_2_ using the 3 M scotch (Magic) tape. The heterostructures were assembled using a method similar to that described in ref. 66 in a custom-built microscope and transfer assembly. For parameters similar to those described in ref. 3, we determined the etching rate of BN, in a CHF_3_ and O_2_ plasma, to be 23±2 nm per 60 s (see Supplementary Fig. 1). The device shown in Fig. 3a was fabricated by etching only the top BN (21±3 nm, etched for 60 s). Two layers of PMMA (450 and 950 K) were spin-coated for electron beam lithography and act as masks for metal deposition and etching. Graphene was contacted by thermally evaporating Au (50 nm) or Cr/Au (5/50 nm) at ≲10^−6^ mbar.

### Measurements

Both average resistance and time-dependent noise were measured in a standard low-frequency lock-in technique, with a small source-drain excitation current ∼100 nA to ensure linear transport regime^67^. Background noise was measured simultaneously and was subtracted from total noise to determine the sample noise.

### Data availability

The data that support the findings of this study are available from the corresponding author upon request.

## Additional information

**How to cite this article:** Karnatak, P. *et al*. Current crowding mediated large contact noise in graphene field-effect transistors. *Nat. Commun.* **7,** 13703 doi: 10.1038/ncomms13703 (2016).

**Publisher's note:** Springer Nature remains neutral with regard to jurisdictional claims in published maps and institutional affiliations.

## Supplementary Material

Supplementary Informations Supplementary Figures 1-5, Supplementary Notes 1-3 and Supplementary References.
