# Supplementary material for: Current crowding mediated large contact noise in graphene field-effect transistors
Source: Nat Commun. 2016 Dec 8;7:13703. doi: 10.1038/ncomms13703 (PMC5155149; doi:10.1038/ncomms13703)
Supplement: Supplementary Informations — Supplementary Figures 1-5, Supplementary Notes 1-3 and Supplementary References. [file ncomms13703-s1.pdf]

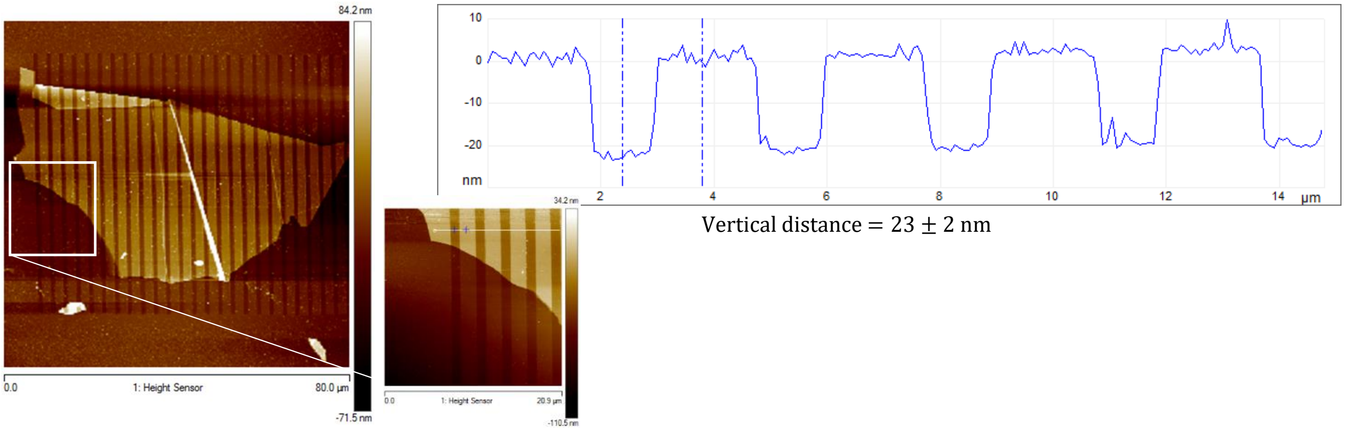

Supplementary Figure 1. Etching depth of BN. BN (height ∼ 40 nm) etches at a rate of ∼ 23 nm per 60s.

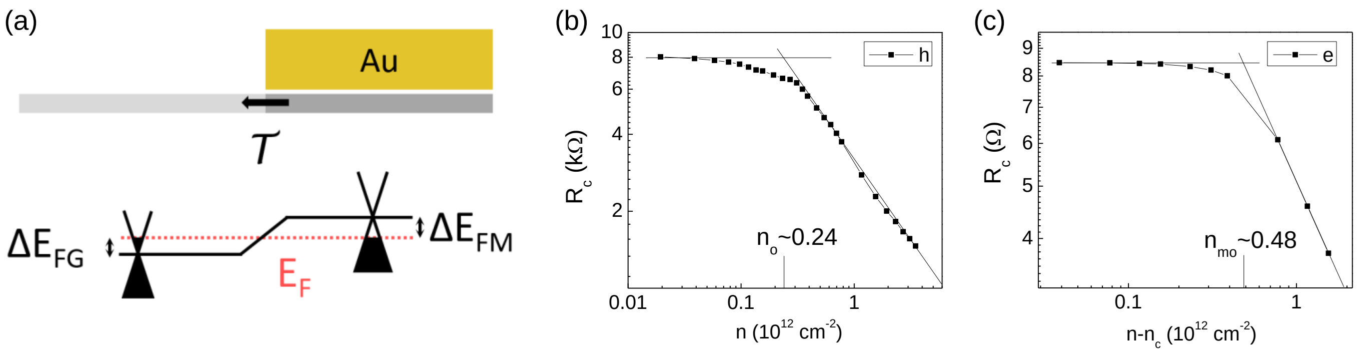

Supplementary Figure 2. Estimating Gaussian widths. (a) Contact resistance across a potential step. (b) and (c) Estimating the minimum carrier concentration in the graphene channel and in graphene under the metal contact respectively.

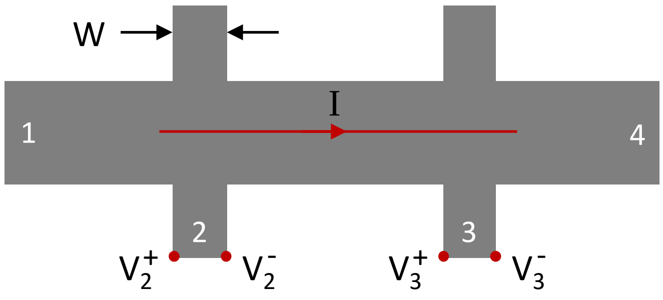

Supplementary Figure 3. Non-local component in a Hall bar geometry.

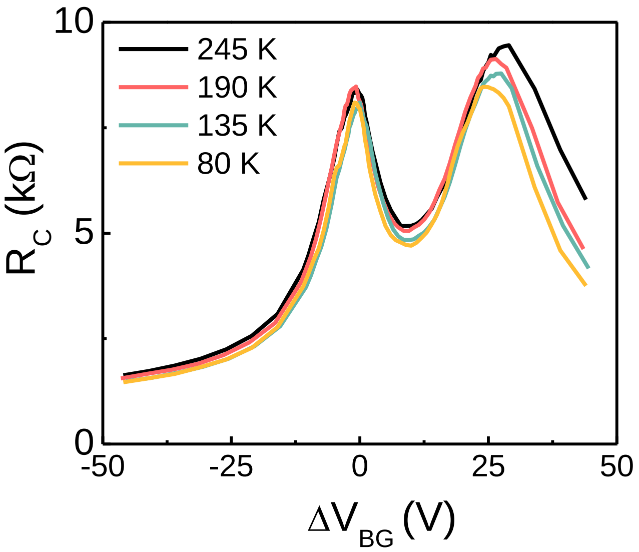

Supplementary Figure 4. Ohmic contacts. The contact resistance is nearly independent of temperature or shows ohmic behaviour for high electron doping (Hall bar device with Au contacts).

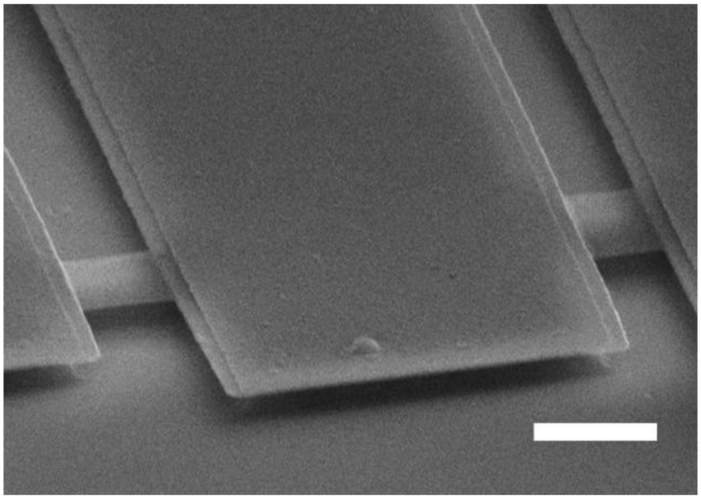

Supplementary Figure 5. Contact noise reduction for suspended graphene. The reduction in contact noise for suspended graphene may occur due to the etching of SiO_2 from under the contacts as well as the entire channel region. This minimises the effect of trapped charge fluctuations from SiO_2.

SUPPLEMENTARY NOTE 1. DEVICE FABRICATION

Hall bar device with graphene on SiO_2 with Au contacts Graphene was exfoliated onto a clean Si/SiO_2 substrate by scotch tape exfoliation technique. Two layers of PMMA (450 K and 950 K) were spin coated on the substrate containing the graphene flake, then electron beam lithography process was used to define contact pads on the flake and subsequently gold (99.999%) was deposited by thermal evaporation. The graphene flake was patterned in Hall probe geometry using electron beam lithography and etched with oxygen plasma.

Hall bar device with graphene encapsulated between two hexagonal Boron Nitride (BN) layers Graphene and BN (hq graphene) were exfoliated on different wafers. Using a transparent polymer layer the top BN was picked up, this

BN under the polymer was then aligned and brought into contact with the graphene to pick it up. The two layer stack was then aligned over the bottom BN and brought into contact. Graphene therefore never comes onto contact with any polymer during device processing. This three layer heterostructure was then transferred onto a pre-patterned substrate for device processing. The heterostructure was etched into a Hall bar geometry using a CHF_3 and O_2 plasma. Another step of lithography defined the contacts and a CHF_3 and O_2 plasma is used to etch the top BN [1]. AFM topographic map determined the top BN to be 21 ± 3 nm and was etched for 1 min. Cr/Au (5 nm/50 nm) contacts are then deposited by thermal evaporation. We determined that BN etches at a rate of 23 ± 2 nm per 60s (Supplementary Figure 1). Hence we do not expect graphene to be fully etched under the contact resulting in a surface contact albeit to a possibly damaged graphene.

Graphene on BN with invasive surface contacts BN (Momentive PT110) was first exfoliated onto Si/SiO_2 with 285 nm oxide. Then graphene was transferred onto the BN following Ref. [2]. The contacts were defined using electron beam lithography and thermal deposition of Cr/Au (5 nm/50 nm). Ar/H_2 annealing and vacuum annealing were performed at different stages of fabrication to remove polymer residues and adsorbates from the graphene surface.

Graphene partially on BN and SiO_2 with invasive surface contacts The single layer graphene was picked up and transferred onto a Si/SiO_2 wafer containing BN, in such a manner that a part of graphene rests on BN and the rest on SiO_2. All processing steps that follow are same for both regions of graphene. Graphene from both regions was patterned into rectangular strips using oxygen plasma. Then electron beam lithography process was used to define invasive surface Cr/Au (5 nm/50 nm) contact pads on graphene.

Suspended graphene The suspended graphene devices were fabricated from standard substrated graphene devices on 300 nm of SiO_2 with Cr/Au (5 nm/50 nm) contacts. To suspend the structure, 200 nm of oxide was etched using a buffered hydrofluoric (BHF) acid solution.

SUPPLEMENTARY NOTE 2. CONTACT RESISTANCE

Gold causes a shift in the work function of graphene underneath, and also leaves the graphene under it largely intact. This allows for the calculation of contact resistance by considering the injection of charge carriers across the potential step (Supplementary Figure 2a), *i.e.* from graphene under the metal with Fermi energy that is shifted from the Dirac point by ΔE_{FM} to the graphene in the channel with Fermi energy shifted by ΔE_{FG}. Since gold does not pin the work function of graphene, both ΔE_{FM} and ΔE_{FG} can be tuned by the back gate voltage, with $\Delta E_{FG} = -\mathrm{sign}(n)\hbar v_F \sqrt{\pi\,|n|}$ and $\Delta E_{FM} = -\mathrm{sign}(n)\hbar v_F \sqrt{\pi\,|n_m|}$.

Assuming ballistic transport across the potential step, the resistance is governed by the minimum number of modes in graphene under the metal contact (M_1) or in the graphene channel (M_2). Disorder potential is assumed to cause a Gaussian broadening (with Gaussian width σ) of the electronic states $G(x,\sigma) = 1/\sqrt{2\pi}\sigma\, exp(-x^2/2\sigma^2)$. The Gaussian widths $t_2 = \hbar v_F \sqrt{\pi n_{mo}} \approx 57$ meV and $t_1 = \hbar v_F \sqrt{\pi n_o} \approx 80$ meV are directly obtained from the experimental data for the main and the secondary Dirac peaks respectively (Supplementary Figure 2b and Supplementary Figure 2c). Assuming the transmission $T \simeq 1$ for all channels, all these considerations allow us to calculate the resistance across the potential step as [3]

$$\frac{1}{R_T} = \frac{4e^2}{h} \int_{-\infty}^{\infty} \mathrm{d}E_1 G(E_1 - \Delta E_{FM},\, t_1) \int_{-\infty}^{\infty} \mathrm{d}E_2 G(E_2 - \Delta E_{FG},\, t_2) \frac{1}{W} \min\{M_1,\, M_2\} \tag{1}$$

without any fitting parameters. R_T is multiplied by a factor of two for a comparison with $R_{2p} - R_{4p}$ (Fig. 1d) due to the presence of two such junctions in the device.

SUPPLEMENTARY NOTE 3. CONTACT NOISE IN A HALL BAR: NON-LOCAL EFFECT

The appearance of contact noise in 4-probe measurements was a surprising observation (Fig. 2b and Fig. 3c). We explain this by considering that a current (I) flowing between the current probes 1 and 4 generates a non-local voltage [4, 5] at the contacts on voltage probes 2 and 3 (see Supplementary Figure 3) and is given by

$$V_2^+ - V_2^- = V_3^+ - V_3^- = IR_{NL} \tag{2}$$

If metal contacts are sufficiently close to the main channel (like the device shown in Fig. 3a) and/or the channel resistance is very small, we have [4, 5]

$$R_{NL} = \frac{\rho_T}{\pi} \ln \left(\frac{\cosh(\pi L_T/W) + 1}{\cosh(\pi L_T/W) - 1} \right) \tag{3}$$

where ρ_T is the specific contact resistance, L_T is the charge transfer length and W is the width of the contact. When $L_T \gg W$ the non-local resistance can be approximated to

$$R_{NL} \approx \frac{4}{\pi} \rho_T \mathrm{e}^{(-\pi L_T/W)} \tag{4}$$

The measured 4-probe resistance is given by

$$R_{4P\_meas} = \frac{V_2 - V_3}{I} = \frac{(V_2^+ + V_2^-) - (V_3^+ + V_3^-)}{2I} \tag{5}$$

$$R_{4P\_meas} = \frac{1}{2I} \left(2V_2^+ - \frac{\rho_{T2}}{\pi} \ln \left(\frac{\cosh(\pi L_T/W) + 1}{\cosh(\pi L_T/W) - 1} \right) I - 2V_3^+ + \frac{\rho_{T3}}{\pi} \ln \left(\frac{\cosh(\pi L_T/W) + 1}{\cosh(\pi L_T/W) - 1} \right) I \right) \tag{6}$$

$$R_{4P\_meas} = R_{4P\_true} - \frac{\rho_{T2}}{2\pi} \ln \left(\frac{\cosh(\pi L_T/W) + 1}{\cosh(\pi L_T/W) - 1} \right) + \frac{\rho_{T3}}{2\pi} \ln \left(\frac{\cosh(\pi L_T/W) + 1}{\cosh(\pi L_T/W) - 1} \right) \tag{7}$$

Since $\rho_{T2} \approx \rho_{T3} (= \rho_T)$, in a time averaged 4-probe resistance measurement the last two terms cancel and $R_{4P\_meas} \approx R_{4P\_true}$. However in a noise measurement the fluctuations in 'resistors' R_{T2} and R_{T3} will add up. The total measured variance in 4-probe is

$$Var(R_{4P\_meas}) = Var(R_{4P\_true}) + \frac{1}{2\pi^2} \left(\frac{W}{L_T} \ln \left[\frac{\cosh(\pi L_T/W) + 1}{\cosh(\pi L_T/W) - 1} \right] \right)^2 Var(R_T) \tag{8}$$

If $Var(R_{4P\_meas}) \sim 0$ we can see that the measured variance in a 4-probe configuration is smaller by a factor η than the contact resistance.

$$\eta = \frac{1}{2\pi^2} \left(\frac{W}{L_T} \ln \left[\frac{\cosh(\pi L_T/W) + 1}{\cosh(\pi L_T/W) - 1} \right] \right)^2 \approx 32 \left(\frac{W}{\pi L_T} \right)^2 \mathrm{e}^{(-2\pi L_T/W)} \tag{9}$$

SUPPLEMENTARY REFERENCES

[1] Wang, L. *et al.* One-dimensional electrical contact to a two-dimensional material. *Science* **342**, 614–617 (2013).

[2] Zomer, P. J., Dash, S. P., Tombros, N. & van Wees, B. J. A transfer technique for high mobility graphene devices on commercially available hexagonal boron nitride. *Appl. Phys. Lett.* **99**, 232104 (2011).

[3] Xia, F., Perebeinos, V., Lin, Y.-m., Wu, Y. & Avouris, P. The origins and limits of metal-graphene junction resistance. *Nat. Nanotechnol.* **6**, 179–184 (2011).

[4] van der PAUW, L. J. A method of measuring specific resistivity and hall effect of discs of arbitrary shape. *Philips Res. Rep.* **13**, 1–9 (1958).

[5] Abanin, D. *et al.* Giant nonlocality near the dirac point in graphene. *Science* **332**, 328–330 (2011).
